# Recurrent, isolated left main coronary artery stenosis in a young female

**DOI:** 10.21542/gcsp.2025.24

**Published:** 2025-05-15

**Authors:** Brianna Skaff, Jeff Spindel, Melissa Miller, Maya Ignaszewski

**Affiliations:** 1 Division of Internal Medicine, University of Kentucky, 800 Rose St, Lexington, KY 40536; 2 Division of Cardiology, Gill Heart and Vascular Institute, University of Kentucky

## Abstract

A 21-year-old female with no prior cardiac history presented multiple times with chest pain and elevated cardiac biomarkers. Single-photon emission computed tomography revealed a reversible perfusion defect, and coronary angiography was attempted, but was aborted. After multiple similar presentations without angiography, the patient suffered ST-elevation myocardial infarction. Coronary angiography revealed 99% subtotal occlusion of the left main coronary artery and a reduced ejection fraction of 20% complicated by cardiac arrest and cardiogenic shock requiring mechanical circulatory support-assisted percutaneous coronary intervention and veno-arterial extracorporeal membrane oxygenation as a bridge to recovery. An extensive workup did not reveal any secondary causes. Despite frequent follow-up and strict adherence to dual antiplatelet therapy, she developed recurrent angina and was found to have severe in-stent restenosis of the left main coronary artery, requiring two-vessel coronary artery bypass grafting. Despite normal blood counts prior to surgery, post-surgical labs revealed blast-phase acute myeloid leukemia, prompting the initiation of chemotherapy.

## Case report

A 21-year-old female with a medical history of papillary thyroid carcinoma after total thyroidectomy (2019), necessitating thyroid hormone replacement, presented to the emergency department (ED) with substernal chest pain that radiated to her jaw with exertion. Physical examination was normal, and vital signs were as follows: heart rate, 72 beats/min; blood pressure, 130/71 mmHg; and respiratory rate, 19 breaths/min. High-sensitivity troponin increased from 143.6 to 294 pg/mL (normal 3-58.8 pg/mL, Abbott Laboratories, Illinois, USA), and there were no ischemic electrocardiogram (EKG) changes. Viral respiratory panel was negative, hemoglobin was 11.6 g/dL and white blood cell count was 9.4 K/µL with 8% monocytes (normal 4–5%) on an otherwise normal manual cell differential. Stress perfusion imaging revealed a reversible defect involving the apex and mid-to-distal anteroseptal, inferior, and lateral walls. Cardiac catheterization was attempted from radial access, but was unsuccessful and aborted. She was discharged home on aspirin 81 mg daily, atorvastatin 40 mg daily, and metoprolol tartrate 12.5 mg twice daily.

**Figure 1.**
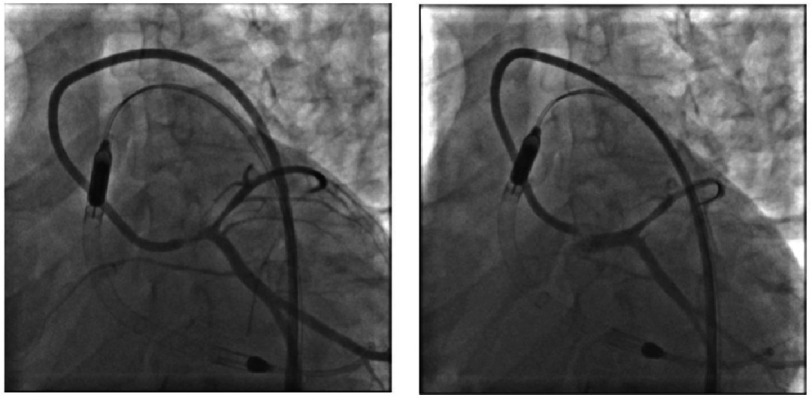
Cardiac arrest presentation. (A) LAO caudal view revealing subtotal LMCA occlusion and left anterior descending artery vasospasm. (B) Same view after balloon dilation.

After returning to work without physical limitations, the patient experienced a syncopal episode associated with substernal chest pain upon awakening approximately two weeks later. The patient presented to the same ED, where EKG was suggestive of anterior STEMI. Immediately before coronary catheterization, the patient experienced cardiac arrest due to polymorphic ventricular tachycardia. Following the return of spontaneous circulation with conventional cardiopulmonary resuscitation, emergent angiography revealed subtotal occlusion of the ostial left main coronary artery (LMCA) (Figure 1).

**Figure 2.**
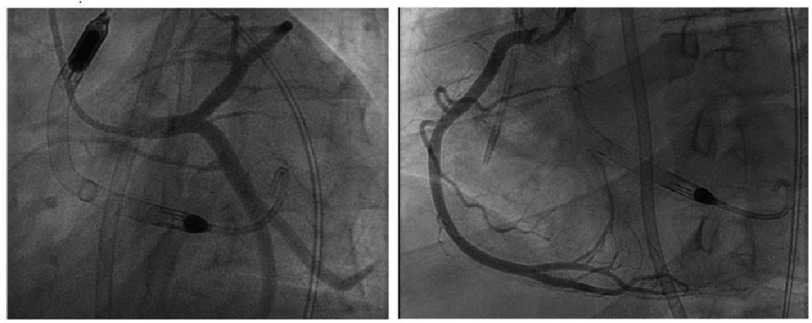
Presentation to referral center immediately after Impella-assisted LMCA PCI. (A) LAO caudal view revealing patent LMCA DES and no evidence of additional atherosclerotic or thrombotic lesions. (B) LAO view of the right coronary artery revealing a normal coronary arteriogram.

Due to worsening hemodynamic instability, a percutaneous left ventricular assist device (Impella CP; Abiomed, Danvers, MA, USA) was placed. LMCA percutaneous coronary intervention (PCI) was performed with suction thrombectomy and placement of a 4.0 x 12 mm Xience drug-eluting stent (DES), post-dilation with a 4.5 mm diameter balloon at 18 atm. (Figure 2). She was transferred to the referral center with Society for Cardiovascular Angiography and Interventions (SCAI) stage D cardiogenic shock and peripheral veno-arterial extracorporeal membranous oxygenation (VA-ECMO) was initiated. Repeat angiography performed immediately preceding cannulation revealed a patent LMCA stent and no additional evidence of coronary artery disease (CAD). Left ventricular ejection fraction (LVEF) on VA-ECMO with Impella CP was 20% with global hypokinesis.

**Table 1:** Lab results for hypercoagulability, autoimmune disorders, malignancy, and endocrine disorders.

**Lab**	**Value**	**Reference Range**
A1c (%)	5.4%	<5.7%
Lipid panel	Cholesterol: 120 mg/dL HDL: 31 mg/dL Triglycerides: 181 mg/dL LDL: 52.8 mg/dL	Cholesterol: <200 mg/dL HDL: >50 mg/dL Triglycerides: <150 mg/dL LDL: <100 mg/dL
Thyroid Stimulating Hormone	0.56 uIU/mL	0.40 –4.20 uIU/mL
T4	1.1 ng/dL	0.8 –1.7 ng/dL
T3	54 ng/dL	87 –187 ng/dL
ESR	31 mm/hr	<20 mm/hr
CRP	173.2 mg/L	<8.0 mg/L
Creatine Kinase	726 U/L	
Homocysteine	6.8 umol/L	5.0 –15.0 umol/L
Cryoglobulin	Negative	–
C3	190 mg/dL	84 –166 mg/dL
C4	31 mg/dL	13-36 mg/dL
Paroxysmal nocturnal hemoglobinuria	No immunophenotypic evidence of paroxysmal nocturnal hemoglobinuria by flow cytometry.	–
ANCA IFA titer	<1:20	<1:20
Myeloperoxidase Ab, IgG	0 AU/mL	0 –19 AU/mL
Serine Proteinase 3 Ab, IgG	0 AU/mL	0 –19 AU/mL
HIV	Nonreactive	–
Hepatitis B Surface Ab	Negative	–
Hepatitis B Core Ab, IgG, IgM	Negative	–
Hepatitis C Surface Ab	Negative	–
ANA	<1:80	<1:80
Anti-Beta 2 glycoprotein, IgG	<1.4 U/mL	<20.0 U/mL
Anti-Beta 2 glycoprotein, IgM	<1.5 U/mL	<20.0 U/mL
ANCA vasculitis	<1:20 AU/mL	<1:20 AU/mL
Cytomegalovirus Ab, IgG	4.70 U/mL	<0.59 U/mL
Ebstein Barr Virus Ab, IgG	64.8 U/mL	0 –21.9 U/mL
Direct antiglobulin test	Negative	–
Glucose-6 phosphate dehydrogenase	14.4 U/g	9.9 –16.6 U/g
Herpes Simplex Virus 1 Ab, IgG	0.29 IV	<0.89 IV
Herpes Simplex Virus 2 Ab, IgG	0.17 IV	<0.89 IV
IgG	850 mg/dL	720 –1,589 mg/dL
Mumps Ab, IgG	Equivocal	–
Rubella Ab, IgG	Positive	–
Rubeola Ab, IgG	Positive	–
Treponema pallidum Ab	Nonreactive	–
Toxoplasma gondii Ab, IgG	<3.0 IU/mL	<7.1 IU/mL
Varicella Zoster Ab, IgG	Negative	–
Strongyloides Ab, IgG	0.4 IV	<0.9 IV
Tetanus Ab, IgG	0.4 IU/mL	<1.0 IU/mL
Quantiferon TB Gold	Negative	–
Lipoprotein A	55 mg/dL	<29 mg/dL
Fibrinogen	586 mg/dL	208 –459 mg/dL
Lactate dehydrogenase	681 U/L	116 –250 U/L
Ferritin	1,188 ng/mL	13 –150 ng/mL
GGT	90 U/L	5 –36 U/L

An extensive investigation was performed to evaluate the underlying causes that predisposed her to the condition. Labs for hemoglobin A1c, HIV, hepatitis B and C, ANA, anti-Beta 2 glycoprotein, homocysteine, schistocyte smear, JAK2 mutation, paroxysmal nocturnal hemoglobinuria, ANCA vasculitis, C4, cryoglobulin, cytomegalovirus, direct antiglobulin test, glucose-6 phosphate dehydrogenase, HLA, HSV, IgG, mumps, rubella, rubeola, syphilis, Toxoplasma gondii, Varicella Zoster, Strongyloides, Tetanus, and Tuberculosis were all unrevealing. Lipoprotein A and C3 were slightly elevated at 55 and 190. Fibrinogen was elevated at 586 and lactate dehydrogenase was 681. Ebstein Barr Virus antibody was elevated at 64.8. GGT was elevated at 90. The patient’s ferritin level was greater than 1,000 (Table 1)

Overall, there was no evidence of hypercoagulability, autoimmune disorders, malignancies, or endocrine disorders. Transthoracic echocardiography with bubble study revealed no evidence of intracardiac shunting. Following clinical stabilization, she was successfully weaned from mechanical circulatory support and eventually discharged to an inpatient physical rehabilitation center with an improved LVEF of 40–45% on aspirin 81 mg daily, ticagrelor 90 mg twice daily, carvedilol 12.5 mg twice daily, spironolactone 25 mg daily, sacubitril-valsartan 24–26 mg twice daily, and dapagliflozin 10 mg daily.

**Figure 3.**
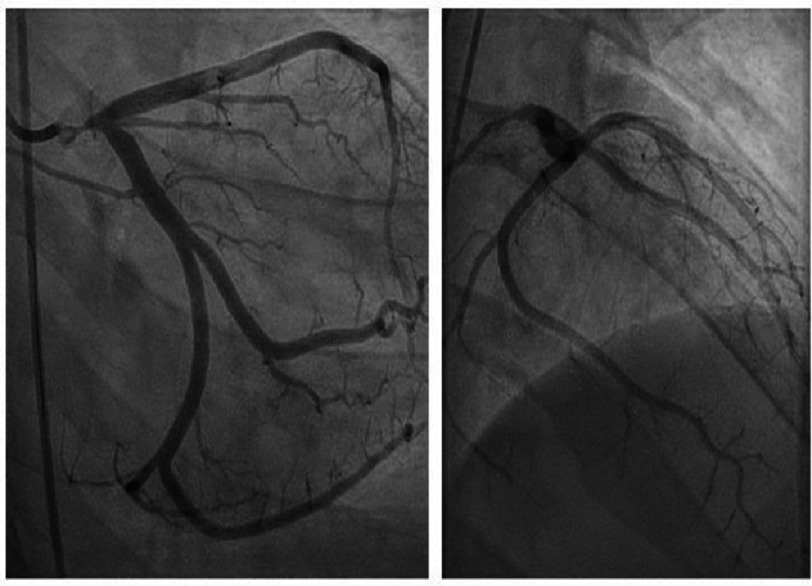
(A) Coronary Angiography, RAO caudal projection of left coronary artery revealing critical LMCA in-stent restenosis. (B) RAO cranial projection.

**Figure 4.**
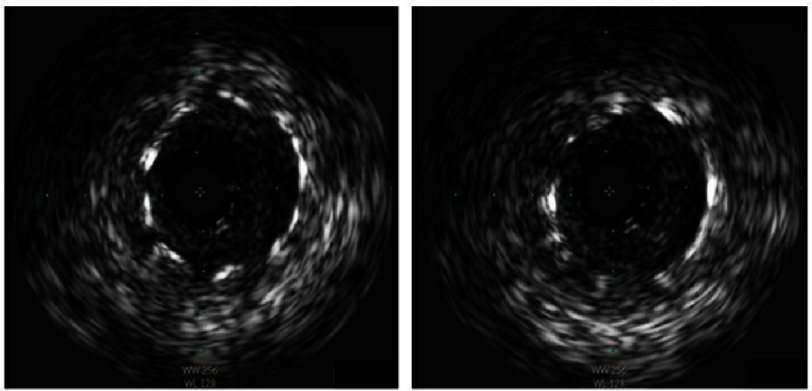
(A) IVUS of proximal LMCA revealing severe in-stent restenosis and neointimal hyperplasia. (B) Distal LMCA.

Seven months later, the patient presented for routine outpatient cardiology follow-up and described symptoms of recurrent exertional angina. Coronary angiography revealed severe in-stent restenosis (Figure 3) with a minimum stent area of 3.4 mm^2^ and severe neointimal hyperplasia based on intravascular ultrasound (IVUS) evaluation (Phillips Healthcare, Andover, Massachusetts, USA) (Figure 4) with a reference vessel diameter of 5.0 mm.

Elective coronary artery bypass grafting (CABG) was performed through median sternotomy using cold blood cardioplegia, with the left internal mammary artery anastomosed to the left anterior descending coronary artery, due to a long LM stent with diffuse stenosis and a saphenous vein graft to the first obtuse marginal artery. Despite normal pre-operative blood work, cell differential of complete blood count three days following bypass surgery revealed 20% acute myeloid blasts and bone marrow biopsy confirmed acute myeloid leukemia (AML). She was treated with Azacitidine and Venetoclax with initial AML remission. She has undergone regular surveillance with biomarkers, ECG, and echocardiograms, and, despite relapse of AML, has not had recurrence of angina or heart failure.

## Discussion

Acute MI and AML are rarely reported as concomitant conditions, however, STEMI with LMCA disease has been seen as the presenting condition that led to diagnosis of AML^1, 2^. One hypothesis as to why CAD is seen in patients with AML is the imbalance between coagulation and thrombolytic factors created by systemic inflammation in this hematologic disorder. The pathophysiology connecting the two disease states includes the formation of intravascular thrombi and a hypercoagulable milieu caused by the release of procoagulant factors from the leukemic cells^3^. Risk factors for stent thrombosis include bifurcation lesions, longer lesions, younger age, resistance or non-compliance with antiplatelet therapy, left ventricular dysfunction, and diabetes^4^. While our patient underwent extensive, unrevealing hypercoagulability testing on several occasions, it is likely that AML contributed to her cardiac presentation, possibly at a pre-malignant phase.

The established incidence of CAD with AML is mostly observed in the older population with multiple comorbid conditions that lead to the development of atherosclerosis, even in the absence of hematologic malignancy^5^. There are few case report ss that include young patients with hematologic malignancies with ACS at ages 9, 15, and 33-years-old^6–8^. However, each patient in these reports had hematologic malignancy diagnosed prior to the development of ACS. Therefore, the cardiotoxic effects of chemotherapy and radiation confound these associations^9^.

We highlight a case of AML associated with isolated LMCA stenosis complicated by cardiogenic shock requiring mechanical circulatory support and development of late in-stent restenosis requiring CABG in a young female patient without concomitant cardiac co-morbidities. While our causative theory is limited by the absence of intravascular imaging at the time of original presentation, this was not clinically feasible due to hemodynamic instability. Furthermore, the chronicity of symptoms and the development of in-stent restenosis of a fully expanded stent lend support to our theory.

### What have we learned?

• All patients, regardless of age or cardiac history, with significant myocardial injury should be further evaluated for underlying cardiovascular diseases.
• Myeloid leukemia has been associated with the development of coronary artery disease; however, most reported cases in the literature were in an older population with other comorbid medical conditions. There are very few cases of younger patients with concomitant hematologic malignancies and ACS. While there are ample studies on the association and prevention of cardiotoxicity from chemotherapy and radiation therapies, there is a lack of data regarding the causation and association of specific malignancies with CAD. Our case highlights the association between two distinct pathologies with limited confounders and requires further research.
